# An Update on Medical and Surgical Treatments of Parkinson’s Disease

**DOI:** 10.14336/AD.2020.1225

**Published:** 2021-07-01

**Authors:** Dipali Nemade, Thyagarajan Subramanian, Vikram Shivkumar

**Affiliations:** ^1^Department of Neurology, Marshall University School of Medicine, Huntington, WV 25701, USA; ^2^Department of Neurology and Neural and Behavioral Sciences, Penn State University College of Medicine, Hershey, PA 17033, USA

**Keywords:** Parkinson, therapies, diagnosis, motor fluctuations

## Abstract

Parkinson’s disease (PD) is characterized by degeneration of dopaminergic neurons in the substantia nigra pars compacta and other neuronal populations. The worldwide prevalence of PD is over 7 million and has been increasing more rapidly than many other neurodegenerative disorders. PD symptoms can be broadly divided into motor (slowness, stiffness, tremor) and non-motor symptoms (such as depression, dementia, psychosis, orthostatic hypotension). Patients can also have prodromal symptoms of rapid eye movement sleep behavior disorder, hyposmia, and constipation. The diagnosis of PD is mainly clinical, but dopamine transporter single-photon emission computed tomography can improve the accuracy of the diagnosis. Dopamine based therapies are used for the treatment of motor symptoms. Non-motor symptoms are treated with other medications such as selective serotonin reuptake inhibitors (depression/anxiety), acetylcholinesterase inhibitors (dementia), and atypical antipsychotics (psychosis). Patients with motor fluctuations or uncontrolled tremor, benefit from deep brain stimulation. Levodopa-carbidopa intestinal gel is an alternative to deep brain stimulation for uncontrolled motor fluctuations. Rehabilitative therapies such as physical, occupational, and speech therapy are important during all stages of the disease. Management of PD is complex but there have been significant advancements in the treatment of motor and non-motor symptoms over the past few years. This review discusses the updates in the medical and surgical management of PD.

## Introduction

Parkinson’s disease (PD) was first described by Dr. James Parkinson in 1817 in his paper titled ‘Essay on the shaking palsy’ [1]. The prevalence of PD has been increasing more rapidly than many other neurodegenerative disorders [2]. The worldwide prevalence is projected to double from 7 million in 2015 to 14 million in 2040 highlighting the enormous burden it poses [3]. The prevalence increases with age and is more common in males than females (1.4:1). About 5-10% of patients with PD have a monogenic form with Mendelian inheritance [4]. The majority of PD cases are sporadic with unknown etiology, possibly caused by an association of genetic and environmental risk factors [4-6]. Among the genetic risk factors for sporadic PD, the most robust and replicable associations have been found for LRRK2, GBA and MAPT [6]. Management of PD is complicated but there have been significant advancements in its treatment. This review discusses the updates in medical and surgical management in PD.

## Methods

A literature search for systematic reviews, national guidelines, and additional articles regarding diagnosis and treatment of PD was performed using PubMed and Cochrane database up to November 2020. We used the search terms “Parkinson’s disease”, “diagnosis”, “treatment”. The searches were focused more on the medical and surgical therapies for PD. Reviews performed within the last 5 years were assigned a higher priority for inclusion.

## Pathophysiology

Parkinson’s disease is characterized by degeneration of dopaminergic neurons in the substantia nigra pars compacta and other neuronal populations [7]. In addition to dopaminergic dysfunction, other neurotransmitters such as acetylcholine, serotonin, and norepinephrine are affected as well [8, 9]. The pathological hallmark of PD is the presence of Lewy Bodies within the degenerating neurons, composed primarily of misfolded alpha-synuclein (α-syn) protein aggregates [10]. A range of non-motor symptoms precede the motor phase of PD including severe constipation [11, 12], delayed gastric emptying, rapid eye movement sleep behavior disorder (RBD) [13] and olfactory dysfunction. Studies show that pathological α-syn is present in the enteric mucosa in early untreated PD [14, 15]. These findings support the Braak hypothesis based upon autopsy studies, that predicted GI symptoms in the pre-motor phase, and that PD spreads in a rostro-caudal manner from the enteric nervous system (ENS) to the central nervous system (CNS) via vagal pathways [16, 17]. Since its publication, Braak’s hypothesis has received critiques (summarized in [18]), and some more recent data to argue that the disease originates in the brain [19] along with a large body of supportive evidence [20-26]. A recent review of autopsy studies from a large series has confirmed the predictions of the Braak hypothesis [27] and also the notion that there could be 2 different subtypes of PD, one that is body-first subtype and another that is brain-first subtype [28]. The consensus in the present literature appears to support the notion that a good majority of ~66% of PD patients present as the body-first subtype with RBD and constipation. Further, a recent study found that constipation and RBD are strongly associated with future decline in some cognitive measures among PD patients, suggesting that early assessment of RBD and constipation may allow better understanding of the progression of cognitive changes in later phases of PD [29].

**Table 1:** Motor and non-motor symptoms of Parkinson’s disease.

Symptom / sign	Description
Motor	
Bradykinesia	Slowness and hypokinesia (progressive reduction in amplitude). Tested in clinic by asking patient to tap index finger and thumb in rapid succession.
Rest tremor	4-6 Hz tremor noted when the limb is at rest. A re-emergent tremor can be present with posture holding (disappears initially with movement but then returns).
Rigidity	Hypertonia that is present in all directions of movement in a joint. It may be accompanied by cogwheeling.
Postural instability	Loss of balance, unrelated to corticospinal, cerebellar, peripheral nerve or other pathology. Usually not seen in early Parkinson's disease.
Others	Hypomimia, hypophonia, dysphagia, decreased arm swing, dystonia
Non-motor	
Neuropsychiatric symptoms	Depression, anxiety, apathy, mild cognitive impairment, dementia, psychosis
Autonomic dysfunction	Constipation, orthostatic hypotension, urinary urgency and frequency, erectile dysfunction, excessive sweating
Sleep dysfunction	RBD, insomnia, daytime sleepiness
Others	Fatigue, hyposmia, aguesia, sialorrhea, pain

RBD: rapid eye movement sleep behavior disorder

## Clinical features

**Figure 1.**
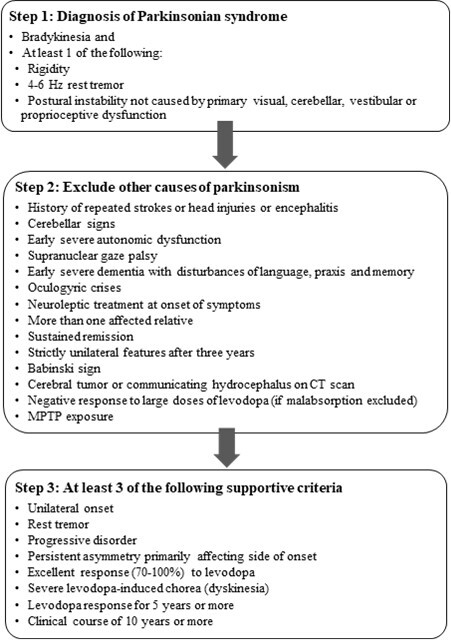
UK Parkinson’s Disease Society Brain Bank Diagnostic Criteria.

The UK Parkinson’s Disease Society Brain Bank diagnostic criteria require the presence of bradykinesia and one of the following features: rigidity, 4-6 Hz rest tremor, or postural instability along with three supportive features (Fig. 1) [30]. The International Parkinson and Movement Disorder Society (MDS) published their diagnostic criteria which include the presence of parkinsonism (bradykinesia along with either rigidity or resting tremor) and two supportive criteria, absence of red flags and exclusion criteria [31]. Postural instability was not included in the MDS criteria since it is usually a late feature of PD and its early occurrence suggests the presence of a Parkinson plus syndrome [32]. A common misconception is that lack of a rest tremor excludes the diagnosis of PD. However, 20% of patients do not present with a rest tremor [33].

PD symptoms can be broadly divided into motor and non-motor symptoms (Table 1) [34]. Patients with PD can have a prodromal period lasting 10-20 years during which they can experience non-motor symptoms such as RBD, anosmia, and constipation. While anosmia and constipation are not specific for PD, they do increase the possibility of future development of PD. Depression has been hypothesized as a possible risk factor or early symptom of PD. However, this remains unresolved at the present time [35]. REM sleep behavior disorder has been shown to be associated with neurodegenerative disorders in over 90% of patients in long-term follow-up [36] and is highly predictive of dopaminergic denervation [37]. A recent study prospectively followed patients with RBD through phenoconversion. Olfactory dysfunction appeared 20 years prior to phenoconversion, impaired color vision, constipation, and erectile dysfunction 10-16 years prior, slight urinary dysfunction and subtle cognitive decline 7 years prior, altered handwriting, turning in bed, walking, salivation, speech, and facial expression 7-11 years prior, and cardinal manifestations of PD such as bradykinesia, rigidity and tremor remained below clinical threshold for diagnosis up to 5 years before final diagnosis [13]

Most patients with PD have a good response to levodopa. Over time, patients can develop motor fluctuations and dyskinesias. Fluctuations are variations in motor symptoms, usually related to the timing of medications but can also be unpredictable. Patients have “off” periods when the effect of the medication wears off resulting in worsening of motor and non-motor symptoms [38]. Dyskinesias are choreoathetoid movements that occur in response to dopaminergic therapy. Motor fluctuations are thought to occur due to certain pathophysiologic changes in the brain [38-40]. The occurrence of dyskinesias has been demonstrated to be linked to disease duration and the dose of Levodopa rather than the duration of exposure to levodopa [41, 42].

## Diagnosis

PD largely remains a clinical diagnosis based on history and examination. Dopamine transporter single-photon emission computed tomography (DaT SPECT) scan is abnormal in PD and other neurodegenerative parkinsonism. It has very high sensitivity and specificity (>98%) for the detection of parkinsonism [43]. The scan is normal in diseases without nigrostriatal cell loss (e.g., Essential tremor, psychogenic parkinsonism, and drug-induced parkinsonism). However, DaT scans are not routinely necessary and are of value only if the diagnosis is uncertain [44]. DaT SPECT does not differentiate between PD and other causes of parkinsonism such as progressive supranuclear palsy (PSP), multiple system atrophy (MSA) and Dementia with Lewy bodies. Despite many advances in research imaging, routine magnetic resonance imaging (MRI) does not show any finding specific for PD [45]. However, MRI may help distinguish PD from vascular parkinsonism and normal pressure hydrocephalus. MRI might also show changes consistent with PSP or MSA in more advanced disease but is not very helpful in early parkinsonism.

## Medical therapies for motor symptoms

### Levodopa

Levodopa is the most potent medication for the treatment of motor symptoms of PD. It has a short half-life of about 1.5 hours and is quickly and extensively metabolized [46]. Levodopa is actively absorbed in the proximal small intestine and is then metabolized by aromatic L-amino acid decarboxylase (AADC). Therefore, levodopa products are combined with an AADC inhibitor such as carbidopa (CD), to prevent peripheral metabolism to dopamine. This ensures better CNS penetration and reduces the risk of nausea. The recommended ratio of carbidopa:levodopa is 1:4, but studies have also shown that a higher amount of carbidopa can increase “on” time without dyskinesias and reduce “off” time [47]. It is best taken on an empty stomach (unless the patient has nausea), at an initial dose of 3-4 times per day at 4-hour intervals during waking hours. The most common side effects are nausea and rarely vomiting in early disease and initial treatment. In more advanced PD, levodopa treatment can cause orthostatic hypotension, sedation, confusion, sleep disturbance, hallucinations and dyskinesias.

Despite the well-documented potency and safety of levodopa, there has been a “levodopa phobia” over the years [48]. Therefore, many patients and physicians have been reluctant to initiate levodopa, especially in cases of young-onset PD. Younger patients are at a higher risk of dyskinesias and motor fluctuations [49]. However, most young-onset PD patients are the ones who need higher potency anti-PD medications as they are functioning at the highest levels raising families and holding jobs. Hence under medicating them with low potency dopamine agonists might condemn them to either give up working or choosing other occupations. Moreover, there is no evidence that levodopa accelerates disease progression or that delaying the use of levodopa prevents dyskinesias [50]. The PD-MED trial showed that patients randomized to early treatment with levodopa (n=528) had a better quality of life (despite dyskinesias) compared to the levodopa-sparing group [51]. Besides, a higher proportion of patients in the dopamine agonist group (28%) and the monoamine oxidase B inhibitor group (23%) withdrew due to adverse effects compared to the levodopa group (2%). The Earlier versus Later Levodopa Therapy in Parkinson Disease (ELLDOPA) study also showed that levodopa does not have any clinical toxicity or neuroprotective effect [52]. The recent delayed-start trial of levodopa showed that maintaining patients on carbidopa/levodopa 25/100 three times a day for up to 80 weeks did not cause more dyskinesias than those who were placed on placebo for the first 40 weeks and then switched to carbidopa/levodopa [53].

Carbidopa-levodopa is also available in other formulations such as the extended-release capsules (IPX066/RYTARY). IPX066 has been shown to be efficacious in reducing “off” time and motor fluctuations [54, 55]. Adverse effects are similar to other levodopa formulations. An inhalable formulation of levodopa was approved by the US Food and Drug Administration (FDA) in 2018 as rescue therapy for “off” periods. It is administered via a breath-actuated device [56]. It has been shown to improve motor symptoms within 10 minutes, achieve maximal effect in about 30 minutes, and last >60 minutes. About 15% of subjects on the drug had a cough which was not dose-related compared to placebo (2%) [57].

### Dopamine agonists

Dopamine agonists (DA) exert their action by stimulating dopaminergic receptors. The agonists currently used are non-ergot derivatives, namely, ropinirole, pramipexole, rotigotine, and apomorphine. DA are primarily used in the earlier stages of PD and as adjunct therapies. Ropinirole and pramipexole are also available in extended-release (ER) formulations. Ropinirole ER is more efficacious compared to its immediate-release (IR) formulation [58]. While pramipexole ER would still have the benefits of more convenient dosing, no difference was noted in tolerability compared to the IR formulation [59]. Rotigotine is administered via a transdermal patch. In comparison to ropinirole and pramipexole, it also has some action on D1 receptors which might confer some additional benefit [60].

The most common side effects of DA include hallucinations, orthostatic hypotension, nausea, pedal edema, excessive daytime sleepiness, and impulse control disorders (ICD). These side effects are more common with DA than levodopa. Patients and caregivers should be warned about ICDs (hypersexuality, pathological gambling, compulsive shopping) prior to initiation and monitored for subsequently at each visit [61]. While reduction or cessation of DA ameliorates these symptoms, some patients may experience a dopamine agonist withdrawal syndrome (DAWS). This is characterized by anxiety, panic, agoraphobia, fatigue, dysphoria, and suicidal ideation [62]. These symptoms do not resolve with the addition of levodopa and resumption of the agonist might be the only solution [63].

Apomorphine is currently available in the United States of America as a subcutaneous injector pen and a sublingual film. Subcutaneous apomorphine is effective for the treatment of morning off periods as well as other daytime off periods [64, 65]. Sublingual apomorphine has been shown to take effect within 10-20 minutes and demonstrated a clinically meaningful and significant improvement in the Movement Disorder Society - Unified Parkinson’s Disease Rating Scale (MDS-UPDRS) III scores [66]. However, 28% (n=15) of subjects on the drug discontinued it due to adverse events with oropharyngeal adverse events being the most common cause (17%). Nausea is an often-bothersome side effect, necessitating pre-treatment with an antiemetic such as trimethobenzamide.

### Catechol-o-methyltransferase (COMT) inhibitors

Levodopa is metabolized peripherally and centrally by COMT. COMT inhibitors thus prolong the action of levodopa and are useful as adjuncts for the treatment of “off” periods. Entacapone is taken as a 200 mg dose with each dose of levodopa. It is also available in combined formulations with carbidopa-levodopa which can reduce pill burden, but clinical outcome is similar. The STRIDE-PD study showed that the use of entacapone in early PD was associated with a shorter time to onset and a higher frequency of dyskinesias compared to carbidopa-levodopa alone [67]. Tolcapone has higher potency due to additional central action. However, associated hepatotoxicity has limited its clinical use. Opicapone is a newer COMT inhibitor that is administered once daily (50 mg). In Phase III studies, it demonstrated superiority over placebo and non-inferiority to entacapone [68, 69]. A subsequent follow-up extension study showed that switching from entacapone to opicapone led to a significant reduction in “off” time and an increase in “on” time without dyskinesia suggesting superiority over entacapone [70].

### Monoamine oxidase-B (MAO-B) inhibitors

Monoamine oxidase is a widely distributed mitochondrial enzyme which catalyzes the oxidative deamination of a variety of monoamines. It has 2 isoforms MAO-A and MAO-B. MAO-A has higher affinity for noradrenaline and serotonin while MAO-B has affinity for beta-phenylethylamine. Tyramine and dopamine are metabolized by MAO-A and MAO-B. Selegiline and rasagiline are selective MAO-B inhibitors used in early PD as well as those with motor fluctuations, but these are generally weak agents. These drugs reduce the degradation of dopamine thus increasing its CNS concentrations. Selegiline is dosed in the morning and afternoon because it has amphetamine-like metabolites which can cause insomnia. A study which evaluated whether selegiline had neuroprotective effects showed that patients treated with selegiline needed levodopa later than the control group, suggesting disease modification properties [71]. However, the results were confounded by the drug’s mild antiparkinsonian and anti-depressant effects [72]. Therefore, it does not have a neuroprotective indication. The PRESTO and LARGO studies demonstrated that rasagiline reduced off time by ~0.9 hours vs placebo [73, 74]. The ADAGIO (Attenuation of Disease Progression with Azilect Given Once-daily) study assessed if rasagiline has any disease modification properties. Though the 1 mg dose group seemed to have a slower rate of progression of UPDRS scores, the 2 mg group showed no such benefit [75]. Therefore, rasagiline has no current role as a disease-modifying treatment. Safinamide is a newer MAO inhibitor that also blocks voltage-gated sodium channels and calcium channels, reducing glutamate release and transmission [76]. It is given once daily (50-100 mg/day) and increases mean “on” time without troublesome dyskinesias [77].

### Anticholinergics

Anticholinergics have been used for the treatment of PD even before the advent of levodopa and dopamine agonists. Benztropine and trihexyphenidyl antagonize acetylcholine at muscarinic receptors postsynaptic to striatal interneurons. They are used primarily for the treatment of tremor in PD. However, they are associated with various adverse events such as confusion, hallucinations, constipation, urinary retention, and dry mouth. There is compelling evidence that the long-term use of anticholinergics in PD contributes to dementia even in younger PD patients [78, 79]. Therefore, these medications have limited use in modern clinical practice.

### Amantadine

Amantadine has been used for a very long time for symptomatic amelioration of PD [80]. However, this symptomatic benefit is of limited value in early PD [81]. Amantadine is often used to treat dyskinesias because of its antiglutamatergic property. It is also thought to block dopamine reuptake, stimulate the release of endogenous stored dopamine, and has a mild anticholinergic effect. Amantadine can cause side effects such as hallucinations and blurry vision (due to corneal edema, a rare ophthalmological emergency). Its dose needs to be adjusted in patients with renal insufficiency. An extended-release formulation (ADS-5102 or GOCOVRI) has been approved for the treatment of levodopa-induced dyskinesias in PD. It is administered orally daily at bedtime. The concentration increases slowly during sleep, attains peak concentration in the morning with sustained levels during waking hours. At the recommended daily dose of 274 mg HS, it results in a 1.4- to 2-fold higher plasma amantadine concentration during the daytime compared to IR formulations [82]. It is available as 68.5 mg and 137 mg capsules. In a phase 3, randomized, double-blind clinical trial it was shown to significantly reduce dyskinesias and “off” time [83]. Another ER formulation OSMOLEX ER was approved for the treatment of PD but the approval was based only on bioavailability studies comparing it to Amantadine IR.

### Adenosine A2 receptor antagonists

Istradefylline is an A2A adenosine receptor antagonist that has been approved by the FDA for motor fluctuations. It reduces the excitability of the indirect pathway by modulating GABAergic transmission [84]. It is available in 20 mg and 40 mg formulations and is administered once daily. It has been shown to reduce “off” time by about 0.7 hours compared to placebo [85]. A post-marketing surveillance study showed improvement in “off” time in 40% of patients [86]. Dyskinesias, hallucinations, nausea, dizziness are the most common side effects.

### Botulinum toxin

Botulinum toxin inhibits the release of acetylcholine from the presynaptic terminals by affecting SNARE and SNAP proteins [87]. Currently, four different preparations are FDA approved in the USA, including onabotulinum toxin A (Botox), abobotulinum toxin A (Dysport), incobotulinum toxin A (Xeomin), and rimabotulinum toxin B (Myobloc). It can be used to treat various symptoms associated with advanced PD. Sialorrhea can be observed in 50% of patients with advanced PD. It is thought to occur due to oropharyngeal dysphagia from bradykinesia rather than over-production of saliva. It can increase the risk of aspiration. Anticholinergics can be used but can lead to side effects. Botulinum toxin injected into the parotid and submandibular glands is beneficial [88, 89]. Dystonia can occur in about 30% of patients and may be more likely in younger patients. Dystonia can occur in the “off” state as well as the “on” state. Botulinum toxin can result in improvement of dystonia and has also been shown to be effective for striatal limb deformities [90, 91].

Botulinum toxin can be potentially injected for refractory tremor, but its use is often limited due to causation of limb weakness. Patients with PD can experience camptocormia which is characterized by involuntary axial flexion while upright. There are a few reports of improvement with injections into the rectus abdominus, but efficacy has been variable [92, 93]. Neurogenic overactive bladder is commonly seen in PD. The need to make frequent trips to the bathroom at night increases the risk of falls. Anticholinergics can result in side effects in older patients. Botulinum toxin injection into the detrusor muscles is effective and is currently approved for the treatment of neurogenic bladder symptoms [94].

The drugs used for the treatment of motor symptoms in PD are summarized in Table 2 based on the 2018 recommendations from the International Parkinson and movement disorders society [95].

**Table 2:** List of drugs for motor symptoms of Parkinson’s disease.

Category	Drug	Clinically useful / possibly useful		Common side effects (excluding dyskinesias); Tips
		Monotherapy or adjunct to levodopa	Motor fluctuations	
Levodopa	Levodopa IR	✓	✓	nausea, hallucinations; Recommended ratio of carbidopa:levodopa is 1:4
	Levodopa CR	✓	✓	
	Levodopa ER (IPX066)	✓	✓	
	Levodopa gel intestinal infusion		✓	nausea, infections, abdominal pain
	Levodopa inhalation powder		✓ (rescue)	cough, nausea, hallucinations
Dopamine agonists	Ropinirole (IR and ER)	✓	✓	nausea, hallucinations, drowsiness
	Pramipexole (IR, ER)	✓	✓	
	Rotigotine patch	✓	✓	nausea, hallucinations, drowsiness, skin site reaction
	Apomorphine injection		✓ (rescue)	nausea, injection site reaction; pre-treatment with antiemetic
	Apomorphine sublingual		✓ (rescue)	nausea; pre-treatment with antiemetic
MAO-B inhibitors	Selegiline	✓	✓	dizziness, insomnia, nausea
	Rasagiline	✓	✓	dizziness, nausea
	Safinamide		✓	dizziness, nausea
COMT inhibitors	Entacapone		✓	nausea, diarrhea, orange discoloration of urine
	Tolcapone		✓	nausea, diarrhea, orange discoloration of urine, hepatotoxicity
	Opicapone		✓	orthostatic hypotension, dizziness
Others	Anticholinergics (e.g., trihexyphenidyl, benztropine)	✓ (rest tremor)		dry mouth, blurry vision, urinary retention; can cause confusion, hallucinations especially in elderly
	Amantadine (IR) and Amantadine ER	✓	✓ (dyskinesias)	pedal edema, hallucinations; dose reduction needed if there is renal impairment
	Istradefylline		✓	nausea, dizziness, hallucinations; avoid in severe hepatic impairment

IR: immediate release, CR: controlled release, ER: extended-release, MAO-B: monoamine oxidase-B, COMT: catechol-O-methyltransferase

## Strategies for treatment of motor symptoms

Patients with minimal symptoms can be treated with MAO-B inhibitors. Young patients who only have a bothersome tremor can be treated with anticholinergics. However, as discussed earlier, these drugs are more likely to cause adverse effects in the long-term and are not recommended. If the patient has significant bradykinesia, then levodopa preparations or dopamine agonists are the drugs of choice. As the motor symptoms progress, patients will need a higher dose of levodopa or dopamine agonists. When they develop “off” periods, in addition to optimizing the above medications, COMT inhibitors, extended-release levodopa, istradefylline, amantadine, and rescue medications such as apomorphine (injection and sublingual film) and inhaled levodopa can be used. Extended-release Amantadine can be used for treating refractory drug induced dyskinesias if fractionation of levodopa is not effective. If motor fluctuations are not adequately managed with the above medications, patients can then be referred for advanced therapies such as deep brain stimulation or levodopa-carbidopa intestinal gel infusion via the placement of an intrajejunal tube and an external pump. It is to be noted that the above strategies are suggestions and there exists considerable heterogeneity in individual approaches to treating PD.

## Non-pharmacological measures

Physical, occupational and speech therapy play an important role in the management of PD [95, 96]. In addition, other non-pharmacological therapies such as tai chi, music-based therapy have also shown benefit [97, 98]. Patients with PD can experience freezing of gait which might respond to levodopa in some cases but is often resistant to medications. In such situations, in addition to physical therapy, the utilization of sensory cues such as lasers and metronomes might be helpful [99, 100].

## Therapies for non-motor symptoms

Patients with PD can develop various non-motor symptoms over the years such as depression, anxiety, dementia, psychosis, dysautonomia (orthostatic hypotension, constipation, drooling), insomnia, REM sleep behavior disorder, fatigue [101]. Non-motor symptoms can often be more disabling than motor symptoms. Cholinesterase inhibitors such as rivastigmine and donepezil and NMDA receptor antagonists (memantine) can be used for dementia. Of these, rivastigmine has greater evidence of being clinically useful [101, 102]. Hallucinations can be associated with PD dementia or anti-PD medications. Amantadine, anticholinergics and dopamine agonists are the most likely medications that can cause hallucinations. The occurrence of psychosis might warrant a reduction in some medications, but this might not be feasible since motor symptoms might worsen. The drugs most commonly used to treat psychosis include quetiapine, pimavanserin, and clozapine. Typical and other atypical antipsychotics are associated with worsening of parkinsonism and should be avoided. Clozapine has been designated as clinically useful and efficacious but requires regular monitoring for agranulocytosis [101]. Quetiapine has lesser evidence of efficacy but is the most convenient to prescribe and therefore, is commonly used in practice. It is often associated with drowsiness due to antihistaminic effects. Pimavanserin was approved by the FDA in 2016 for the treatment of hallucinations and delusions in PD [103]. It is a selective serotonin 5-HT2A inverse agonist and is administered as a 34 mg capsule once daily. It does not typically result in drowsiness since it does not have antihistaminic effects. These medications used for psychosis are associated with a black box warning regarding their use in those with dementia.

Selective serotonin reuptake inhibitors and selective serotonin-norepinephrine reuptake inhibitors along with cognitive behavioral therapy are used for the treatment of depression and anxiety. Pramipexole has also been shown to be efficacious [101]. Fatigue and daytime drowsiness are difficult to treat but modafinil and methylphenidate can be tried. REM sleep behavior disorder can be treated with melatonin or clonazepam. Insomnia is treated using melatonin, hypnotics, trazodone, mirtazapine, and quetiapine.

Orthostatic hypotension can be managed with non-pharmacological measures such as increasing water and salt intake, and compression stockings. Patients should be educated to change positions gradually. Eating smaller, more frequent meals, and reducing carbohydrates can reduce postprandial hypotension [104]. Patients should be educated about the diuretic effects of caffeine and alcohol. Diuretics, vasodilators, and even levodopa and dopamine agonists can worsen orthostatic hypotension. Medication options for hypotension include fludrocortisone, midodrine, and droxidopa. These medications can lead to supine hypertension, so patients should be instructed to not lay supine. They should ideally sleep with the head-end of the bed elevated 30-45 degrees. Use of pressors such as droxidopa and midodrine should be avoided closer to bedtime. Probiotics, fibers, stool softeners, laxatives, lubiprostone, and linaclotide can be used for constipation [104].

## Surgical therapies

Despite multiple medication options, some patients continue to experience a prominent tremor or uncontrolled motor fluctuations which significantly impact their quality of life. In such situations, there are surgical options that might provide relief. However, the exact time when such surgical therapies need to be implemented can be controversial.

### Deep brain stimulation (DBS)

DBS involves surgical placement of leads unilaterally or bilaterally in the subthalamic nucleus (STN) or globus pallidus interna (GPi). These leads are then connected to an implantable pulse generator (IPG) which is usually placed in the chest. The exact mechanism of action is unclear, but it is thought to modulate pathologic firing patterns in the cortico-basal ganglia network [105, 106]. The primary indications are uncontrolled motor fluctuations, dyskinesias, or tremors. It is important to note that, except for tremor, DBS can improve only symptoms responsive to dopaminergic therapy. Compared to best medical therapy, DBS improves “on” time without troublesome dyskinesias [107, 108]. Patients tend to experience the benefits of DBS for even more than 10 years though some studies have reported that its effects on bradykinesia and rigidity may be limited to 5 years [109, 110].

One common question is whether DBS should be performed at an earlier stage of PD or at a younger age. Later in the course of the disease, several motor symptoms tend to be unresponsive to dopaminergic therapy. Therefore, optimizing the quality of life when patients are more responsive to dopaminergic therapy is the ideal approach. In a randomized trial of 251 patients (mean age of 52 years; mean duration of PD 7.5 years) with early motor complications (EARLYSTIM), STN DBS was compared to medical therapy alone [111]. The mean quality of life score improved by 7.8 points in the DBS group compared to 0.2-point worsening in the medical group. It showed that neurostimulation was superior with respect to mobility, levodopa-induced motor complications, motor disability, and activities of daily living [111].

The subthalamic nucleus and globus pallidus interna are the most commonly targeted structures in PD. The superiority of one over the other has long been debated [112, 113]. Another randomized trial comparing the outcomes between subthalamic and pallidal stimulation showed no difference in motor improvement as determined by UPDRS III scores [114]. Subthalamic stimulation is more likely to allow a reduction in dopaminergic medications. Stimulation parameters are usually lower, thus allowing for longer intervals between IPG replacements. STN stimulation might be associated with a greater decline in mood and cognition compared to GPi. Reduction of dyskinesias has been reported to be greater with GPi though reduction of medications post STN DBS might also alleviate dyskinesias. The ventral intermediate nucleus (VIM) of the thalamus can be targeted too. It is only effective for tremors, not other PD symptoms, and therefore is not used commonly.

The major contraindications would include dementia, levodopa unresponsive symptoms (e.g., gait disturbance), and unstable psychiatric conditions. The rates of common surgical complications are variable in literature [115]. An analysis of over 1000 patients showed that rates of infection were (2-3%), while others were intracranial hemorrhage (<2%), lead erosion (1%), lead migration (1%) [116]. Stimulation related side effects include paresthesias, dysarthria, diplopia, gait impairment, depression, hypomania, and cognitive decline [116].

There are three manufacturers with approved devices in the USA [117, 118]. Medtronic was approved for use in PD in 2002, the Abbott Infinity system was approved in 2016 followed by the approval of the Boston Scientific Vercise system. The Medtronic IPGs are Activa PC (Primary Cell), Activa RC (rechargeable), and the recent Percept PC, which has Brainsense technology to track patient-specific brain signals and correlate these to symptoms and side effects. The Medtronic lead has 4 contacts and does not offer directional stimulation. It is approved for whole-body MRI when certain conditions are met. The Abbott IPG is non-rechargeable while the lead is segmented (1-3-3-1 configuration) and allows directional stimulation. It is MRI compatible provided certain conditions are met. The Boston Scientific system has the Vercise PC and Vercise Gevia (rechargeable) IPGs. The lead is segmented (1-3-3-1) but allows for multiple independent current control. The Vercise Gevia has conditional MRI approval but the Vercise PC does not. The factors that contribute towards the choice of the system include the anatomical target, surgeon and neurologist preferences, need for rechargeable IPG, MRI compatibility, and patient preference.

### Levodopa-carbidopa intestinal gel (LCIG)

LCIG is administered via percutaneous endoscopic gastrostomy with a jejunal extension tube (PEG-J). It is delivered by an external pump that administers small doses of levodopa/carbidopa about once every minute to the small intestine [119]. This bypasses gastric emptying, which is often responsible for irregular absorption of levodopa. LCIG provides stable concentrations of levodopa throughout the day [120]. It is indicated in patients with severe motor fluctuations when other medication options have not yielded satisfactory results. A meta-analysis of LCIG showed that it reduces off time by 1.19 hours per day and increases on-time without troublesome dyskinesias by 0.55 hours per day [121].

Few open-label studies have reported improvement in the Non-Motor Symptom Scale (NMSS) with the use of LCIG [122, 123]. However, there are no randomized controlled trials with non-motor symptoms as the primary endpoint. The most common procedure-related adverse events are dislocation of the tube, infection, peritonitis, pneumoperitoneum, obstruction of the tube, erythema at stoma, abdominal pain, and pump malfunction [119]. Side effects related to LCIG infusion include nausea, dyskinesias, and hallucinations. Peripheral neuropathy has been reported as a side effect of LCIG therapy [124]. The etiology is unclear, but it has been suggested that this might be related to vitamin B12/folate deficiency. Therefore, these should be monitored and/or supplemented during LCIG therapy.

There are no randomized controlled trials comparing LCIG and DBS. A meta-analysis reported that LCIG and DBS have similar efficacy in the improvement of motor function [125]. An open-label, non-randomized study has shown that in comparison to STN DBS, PD patients treated with LCIG might significantly improve some neuropsychological functions such as delayed recall, recognition, learning, and visuospatial function [126]. The study also did not find any significant cognitive or behavioral changes in patients treated with STN DBS compared to those receiving conventional medical treatment. However, due to the substantial risks associated with LCIG, it might be preferred over DBS only in older patients with more cognitive deficits.

### Focused ultrasound (FUS)

FUS involves the precise, incision-less, transcranial delivery of acoustic energy. Patients are placed in a stereotactic head frame which is coupled to an MRI-compatible ultrasound transducer. The acoustic energy is titrated to attain temperatures sufficient for tissue ablation. Lesioning can be monitored by magnetic resonance thermometry. A randomized controlled trial of 27 patients (of whom 20 underwent unilateral FUS while others underwent sham procedures) showed a 62% improvement in Clinical Rating Scale for Tremor (CRST) scores [127]. The most common adverse events in the study were paresthesias and ataxia which were permanent in 20% and 4% of patients, respectively. The rates of adverse events were similar to another study of FUS thalamotomy in essential tremor patients [128]. Contralateral weakness, dysphagia, and dysarthria are other potential adverse effects of lesioning. Headache, nausea, and dizziness are the most likely intraprocedural adverse events which usually resolve.

FUS is only performed unilaterally due to the risk of speech and gait disturbances. Currently, FUS is FDA approved in the USA for only unilateral thalamotomy in PD patients. Thalamic targeting, while useful for the treatment of tremor in PD, is ineffective for other motor symptoms of PD. FUS unilateral subthalamotomy has been shown to improve rigidity and akinesia in addition to tremor [129]. However, randomized controlled trials are needed to support the findings. Thus, the current applicability of FUS in PD is very limited and may only be used as a palliative measure for tremor reduction in those with advanced PD who are not candidates for more invasive procedures such as DBS [127].

## Conclusion

Parkinson’s disease is a complex disease with motor and non-motor symptoms. Management involves the use of combinations of various pharmacological and non-pharmacological measures as well as device-assisted therapies in advanced stages.
